# Discussions in Homœopathic Societies and Treatment of Intermittent Fever

**Published:** 1882-07

**Authors:** P. P. Wells

**Affiliations:** Brooklyn, N. Y.


### HOMŒOPATHIC PHYSICIAN.

A MONTHLY JOURNAL OF MEDICAL SCIENCE.

"If our school ever gives up the strict inductive method of Hahnemann, we are lost, and deserve only to be mentioned as a caricature, in the history of medicine."—CONSTANTINE HERING.

Vol. II. JULY, 1882. No. 7.

### DISCUSSIONS IN OUR HOMŒOPATHIC MEDICAL SOCIETIES, AND TREATMENT OF INTERMITTENT FEVER.

P. P. WELLS, M. D., BROOKLYN, N. Y.

Our homœopathic medical societies, national, state and sectional, are supposed to have their uses and their duties. By association, it has been thought, combined effort might give greater force and efficacy to endeavors to increase knowledge of the elementary truths of the philosophy we profess to practice, and a more ready apprehension of the methods and means this practice pursues and employs. This increase of knowledge and the greater readiness in its practical application were the principal objectives, or were supposed to be, of the creation of these organizations. Into these were to be brought the mature thoughts, careful observation of facts, and the practical experiences by which this philosophy had been illustrated and its truths confirmed. This object certainly was a worthy one, and in it is all of usefulness and duty we can imagine as pertaining to these organizations. It may be a question whether this object has not, to a great extent, been lost sight of, and in many instances has well nigh disappeared from their history. Is it not rare that the elementary principles of the homœopathic philosophy are intelligently discussed or illustrated in their meetings? Are not these, more than most other questions, distasteful to a majority of the membership of most of them? Is there not rather an intolerance of these, than an earnest interest in them, manifest in most of the papers and discussions which occupy the time and attention of members at their meetings? Has the influence of these meetings, as a whole, been to strengthen the confidence of members in the authority and efficacy of our law? Have they resulted in a clearer perception of its logical corollaries, or in a more loyal obedience to this law, and compliance with its plain, practical rules? Has not the endeavor been oftener to palliate, excuse or advocate transgressions of law, and to publicly disparage the means it employs for its healings? Have we not oftener heard, at these meetings, our materia medica derided and accused of impurities than we have received truthful and helpful additions to its treasures? Has it not been rare, compared with these offenses, that these bodies, so deriding or neglecting our law and its corollaries, and disparaging our materia medica, have contributed anything helping us to a readier or clearer differentiation of remedies in treating the sick? What knowledge of this we have, has been derived from individuals rather than associations. The Bœnninghausens, Haynels, Herings—these are the men who have given us knowledge, and who found our law and its corollaries equal to their needs, and our materia medica a mine of wealth, which they were able to use, and were never found scolding or abusing it.

The discussions in these bodies to which we have listened, or reports of which we have read, have oftener than otherwise been characterized by statements of what members of these bodies have done, in treating certain diseases or epidemics, or of the peculiar views the members may have had of the *rationale* of the points discussed. It has been of the rarest that they have given proofs that these doings have had their foundation on our law, and that their successes, if they had had successes, were the legitimate outcome of a compliance with the requirements of God's law of healing. Indeed, the law, oftener than otherwise, has seemed to be farthest from the thoughts of the occasion. If brought to the surface, it may have been only to express a disgraceful independence of its authority, and an ill-judged determination to set it at naught at their pleasure, putting its sanctions, corollaries and means at the disposition of whatever of whim may for the moment move the prescriber to this erratic course, and all this in the interest of what has been claimed as a rightful personal liberty. They are not to be "pent up" in any little "Utica" of law or logic, not at all. The liberty to do as they please, this they will have, and if law interposes objections to this, they have the recourse of hating law, and they do it, and in this they not unfrequently include all who give allegiance to law as a guide in their practical duties. These become objects of hate, hard names, accusations of bigotry, narrow-mindedness, and fanaticism, or worse. Why those who thus hold all that constitutes Homœopathy a science and guide to healers in such low esteem, if not in aversion, should claim to be ranked as homœopathists, is not very apparent; especially those who are never so active as when engaged in endeavors to destroy it, or whatever of confidence others may have given to it. It cannot be denied that their chief power in the accomplishment of this evil comes from their professed allegiance to this they are so eager to destroy. It is noticeable of most of these discussions that the subject is seldom taken up from its foundation—and if this be a form of disease, that in its elementary phenomena, in which the relationship of curative is to be found in like elementary phenomena of the curing drug, this is seldom brought out. It is the order, rather, that the disease is named, and then treated as if an entity, and if the "like" which cures is at all in question, it is oftener than otherwise this is to be sought in another entity, more or less imaginary, leaving the elements of the case in which the curing relationship really exists out of the question altogether. How such discussions benefit science, or humanity, or add to human intelligence, it is impossible to imagine.

These thoughts have been suggested by a reported and published discussion in the *Hahnemannian Monthly*, for April, 1882. This has been taken as the occasion of these remarks, and the comments we propose to make on some of the practical utterances therein contained, not because in the particulars mentioned as common to such discussions they are more away from the law and destitute of its spirit and regardless of its authority than have been the average of those of which we have had experience, but because it presents a fair example of them, and is the last which has come to our knowledge, and gives an opportunity for some practical remarks which, from the character of this discussion, would seem to be somewhat called for. The subject under consideration which was discussed, and the observations upon it reported, as mentioned, was *Intermittent Fever.*

The first fact which impressed itself on the mind, as the reading of the report progressed, was the general absence of homœopathic or Hahnemannian ideas (which are the same thing), in the view these members of this county society took of disease, as was evinced in their remarks on the treatment of this fever. It seems, from their observations, to have been to many of them a name, and that name a thing, the identity of which was repeated in every new example of it which became a subject of their treatment. This is certainly wholly foreign to homœopathic philosophy, and perfectly characteristic of that of its antique antagonist, allopathy. The doctor who opened the discussion was called to this duty by the president. Being so called upon he could "not be silent. He must speak," and "*even do a little bragging.*" This is how he did it:

* * * "I feel that I have been very successful. I attribute my success entirely to this, that I treat my cases homœopathically, rigidly adhering to the principle, *similia similibus curantur*, in all cases."

It is no wonder then that he was "successful." The man who can well do this, and who does it "in all cases," may not be censured if he "brags a little." And again, "I follow the precepts of Hahnemann and prescribe for the totality of the symptoms. I individualize each case and get all the symptoms together, those which are most prominent and those which are least so, the modalities, *etc.*, and having done this, I choose that which is the homœopathic remedy in the case." This is certainly just the way to do it; but what has been the outcome of all this very difficult proceeding on the part of the doctor? For whoever has done this faithfully has found it a difficulty.

* * * "I find that this (the homœopathic remedy) is in most cases * * * *Quinine!*"

It is difficult to resist the impression that the doctor is largely at fault in his ideas of the "*totality,*" and the "*simillimum,*" and of the analysis and comparison by which this is found, as required by homœopathic law. It is the more difficult to the reader of this statement if he has had a homœopathic experience of his own. It must be admitted, however, that one cannot bring these statements of the doctor to a final judgment in the light of the facts of experience with ague contracted in other localities than the neighborhood of Philadelphia. For it is true that this form of disease differs greatly in its relations to curatives, as its origin may have been in one locality or another. And it is little less true that cases originating in the same locality present great variety in their elements by which the curative relationship is disclosed and dominated. It may not, therefore, be wise or warranted for one not of Philadelphia to charge this experience of the doctor with defect of understanding of law, symptoms, and curing agents and relationships as these are to be understood, in order to a truly homœopathic practice in this and all diseases; still, when one remembers his own experience, not a very limited one, with this "plague of all diseases," he cannot be otherwise than amazed at the doctor's, which has this *one* remedy the simillimum of the totality which, when properly gathered, are found to present so great diversity in successive cases. The writer has been dealing with ague, just in the same manner as our doctor says he has, about thirty-eight years. He has had more to do with it than he has wanted. He has found it more difficult to secure the simillimum for these cases than for most other forms of sickness. And when he remembers the fact that in all these years he has met but three cases in which an intelligent prescriber could find symptoms calling for quinine to the exclusion of other remedies, it is not at all strange if he has suspicion of the accuracy of this doctor's knowledge, or practice as according to our law. Nor is it strange if this suspicion is confirmed by his statement that "sulphate of quinia, Peruvian bark, China, Cinchonidia, and others * * * are nearly identical so far as their pathogenetic or curative effects are concerned." So far as known this is not true of any two drugs that their curing agency is identical with each other. The intimation that this is true of this number of substances is more than a hint of lack of that thorough analysis and comparison a true homœopathic prescription requires, and this hint will be emphasized by the attempt of the doctor to give the "keynotes" of *Nat. mur.* and *Caps.* If this is a specimen of his knowledge of the elements of the actions of these drugs by which they are related to this fever, he has been but poorly furnished, for a comparison of practice with these and other drugs—quinine, for example—with a view to ascertaining the comparative frequency of cases calling for either, or of their comparative power to cure. In the practice of the writer scores of cases have been cured with *Capsicum* to one by either *Quinine* or *China,* and we have no recollection that any one of them received *Caps.* because the chill "began in the back." We fail to find in *Allen's Encyclopædia* any mention of *Herpes labialis* in the pathogenesis of quinine, though the doctor says, "it may be found also" in cases where quinine is the curative. How does he know this? It is difficult to resist the impression after reading these and other utterances of the doctor in this discussion, that he is altogether loose in his views of homœopathic science and practice. Its philosophy contemplates no such thing as a simillimum for a class of sicknesses, but instead of this, it requires of him who will practice it in its integrity, that he shall find it for each successive case he is called to care for.

And then, further, it is worthy of remark that so many of those taking part in this discussion seemed to have in view only the generic symptoms of ague—the chill, the heat and the sweat—and in their ideas of its homœopathic treatment, to be wholly busy in thinking of a simillimum to these. With all deference to these gentlemen, we beg to assure them that the "like" which cures, is not found chiefly, if at all, in these. It is as though their habit has been to give a name and then try for a simillimum of the symptoms which have justified the *name,* in some drug. Again, with deference, we affirm this is not homœopathic practice of medicine. It is to attempt a homœopathic practice from an allopathic standpoint, and to carry it out on allopathic principles. It is to make a diagnosis, and then to find a remedy for the *name.* This is not homœopathic philosophy or practice by whomsoever it may be advocated, but just the opposite of all that is characteristic of both.

The doctor says: "If there be any physician present who has not given Quinine, * * * it is because certain medical popes and bosses have said he must not." We have given it but seldom, as we have said, not because of "popes" or "bosses"—we have no knowledge of these characters or their sayings, but because the drug has *not been called for by our law.* It is the law, and not "popes," to which we have given heed in this omission. And then the wretched results of failures after the use of this drug by others, which have come under our observation, in *many* cases, have not prepared us to listen with much of either respect or patience to the claim set up in this discussion for this drug as an almost universal curative of this often troublesome malady. It was the fortune of the writer, some years ago, to be called to prescribe for a man who had contracted ague in Illinois, three years before, and from which and quinine, of which he had been taking large quantities and in large doses, he had been a constant sufferer, the result of which, when best, was only a "*suppression*" of the paroxysms for a brief time. This man was cured permanently by the first prescription of a really homœopathic remedy. Another. A woman from near Cleveland, Ohio, had suffered from ague twelve months. She had taken all the quinine she could stagger under, and was rather worse than better for it. She determined to take no more, and was cured by a single dose of a remedy which was really homœopathic to her symptoms, which quinine certainly was not.

One word as to this "*suppression*" of ague by quinine. I supposed that the fact that this often results from the use of this drug was generally known and acknowledged. Our doctor rather sneers at the idea—does not believe in it. He insists the drug *cures*, rather. He says: "If I had *suppressed an ague* * * * for 38 years I should feel that I had done a good thing." This could be better judged of if we could have the facts of the history of the health of the patient during these 38 years of "suppression." A suppressed ague is not always a quiet enemy, though its paroxysmal expression has been suspended. In these circumstances it often works great mischiefs and causes much suffering. It became my duty, while visiting a distant city some years ago, to investigate and prescribe for a case of chronic disease of eleven years' standing, the patient being a banker in that city. In these eleven years he said he had "taken medicine enough to stock a drug store." The beginning of trouble was an attack of ague, in the State of Michigan. He was treated (and "*cured*," our doctor would say, perhaps, as he had no paroxysms of ague in all these years) by large doses of quinine. The days of these eleven years had *all* been sick days. After a careful study of the case it became evident to the writer he was suffering from "suppressed" ague and quinine, and he told the sick man this was the matter, and that he would be no better till his ague returned in the form of its original paroxysm. This returned after the first dose of the prescription given, and the patient was then permanently and perfectly and easily cured. Perhaps his original prescriber might have thought he had "done a good thing," as there were no paroxysms of ague in all these years. He might, possibly, have "bragged" of it a "little." How is it that men who make such mischief in their endeavors to cure, do not see the evil of their work? I can see but one explanation of this, the facts are certainly patent enough to any one who will see. I can only explain the fact of any intelligent man failing to see them by supposing him of the number of those of whom it may truthfully be said, "Sin has blinded the minds of them." The kind of prescribing advocated by some who participated in the discussion under our hand, is nothing less than sinning against our law, and, therefore, by the force of moral law, they are blind and cannot see the difference between suppression and cure. And apparently they are equally blind as to the symptoms in which the law finds the simillimum which cures. They seem to have been unable to find these as other than the generic or defining symptoms with which the curing relationship of our law has comparatively but trifling concern. "The symptoms most important to the diagnosis are often of least interest to therapeutics."—*Hering.*

One of the doctors taking part in this discussion of Intermittent Fever, finds his admiration of the element of debate to which greatest attention had been given—quinine—so great that he exclaims: "If I could get along without quinine, as some claim to do [some certainly do], I would get along without Homœopathy altogether." That is, if deprived of *one* remedy, for the treatment of *one* form of disease, for which, at best, we should say, from our own experience, it is but seldom the best remedy, he could give up God's universal law of healing, with its immense provings of drugs, each a possible cure for some other form of disease. We have heard many silly utterances in society debates, but, perhaps, never another quite so silly as this. If this be a fair specimen of the doctor's intelligence, we cannot doubt the world will be the gainer if he will kindly consent to try and "get along without" both. This is greatly confirmed by the attempt he made to show the homœopathicity of a mass of disjointed fragments of symptoms, mostly of a generic character, to this disease, often so difficult to meet with its true specific cure. He appears so utterly ignorant of both as related to specific medicine, that it would seem his greatest service to this can only be found in his compliance with his own hint and give them both up.

No doubt, hygiene is important in treating this fever, as it is in treating all forms of sickness. But to recommend this as a substitute for the specific remedy, or to intimate that it can ever do the work of that remedy, is to make a grave mistake as to the nature of the office of the two agents. The remark of one of the doctors in this discussion to the effect that if he could not have both, he should have a choice as to which he would dispense with, is an illustration of the very general fact of how little of thought and preparation the members of these societies bring to these discussions. We believe we should do this doctor an injustice if we should say he knew no better. It also illustrates the little worth of this class of discussions, because they are oftener than otherwise entered upon without preparation and prosecuted with little regard to fact or logic.

We have suggested an excuse for these blind violators of our law, as possibly found in the *genius loci*, where the malaria originated, causing the cases so uniformly calling for one remedy, and in it finding their cure. We were glad to be able to suggest this as possible, for, otherwise, these doctors who inculcated this quinine practice as an almost universal compliance with the demands of our law, would stand before all enlightened homœopathic experience convicted of ignorance, slovenliness, or laziness, one of these, or all. And to pass such a sentence on one's neighbor can only be disagreeable in the extreme. But this excuse is taken from them hopelessly by the testimony of their neighbors taking part in this discussion. They had found the agues of Philadelphia and its neighborhood varying in their character and calling for varied remedies for their cure, just as intelligent homœopathists have found agues elsewhere. These objectors to an almost exclusive quinine practice talked very like men who knew what they were talking about when speaking of our law, its materia medica, symptoms of disease and drugs, and the relationships of these by which the one is cured by the other. Indeed, so exactly was their experience and testimony like that of all intelligent observers of other places, we cannot but accept their testimony as truth, and look on the malaria and agues of Philadelphia as very like the same disagreeable facts as we have found them elsewhere. If this judgment leaves these advocates of quinine in an unpleasant position, neither we nor our law are responsible for their dilemma. Specifics for classes of diseases are not within the domain of our law. They never have made any part of its promises to the sick of mankind. The idea is foreign to the essential nature of true Homœopathy, and seems to be inherent in that sham pretense of it which is constantly finding out that this, that and the other of diseases will be certainly cured by a certain drug. This is the allopathist's idea of specific medication, not ours.

One participant in this discussion agreed with another member in the judgment, that once invaded by ague malaria, the unfortunate is never free from it after. It is possible these gentlemen are more familiar with this general specific idea and practice than that with the true simillimum. Quinine may, and very often will, fail of this deliverance, because it is not often, certainly in this neighborhood, the simillimum required for this deliverance. Suppose our friend should try this, and see. One case is certainly not enough to *establish* a *principle*, though it may a *possibility*. Thirty-eight years ago the writer was attacked by a chill which lasted three hours. Its violence was so great, it seemed as if all his bones would be broken and his teeth knocked out. This was followed by heat, restlessness and pain, equally excessive, and this by a more moderate sweating. He was obliged to be his own physician. When he had made a point in the symptomatology of the case, he caused it to be written on a slate. When all was gathered, he had his materia medica brought to the side of his bed, and to this he applied himself, when the excessive pain in his head would permit, and after a search of twelve hours he was satisfied he had done his best and believed he had found his needed simillimum, though he had then never heard of this medicine being given for the cure of ague. This made no difference either to his confidence or the cure, as it turned out. He took one dose of this, then stranger to him, and he has never had a chill from that time to this. We believe this answers the question as to the possibility of malaria being eradicated by the specific remedy. It is safe to say that it seldom is by the slip-slop prescribing of which this advocated general resort to quinine is an eminent and reprehensible example.

